# Progress in Medicine

**Published:** 1895-10-26

**Authors:** 


### Progress in Medicine.

### GRAVES' DISEASE.

**Pathology.**—We recently described the pathological changes observed by Edmunds[1] in the thyroid gland in Graves' disease, and the investigations of Notkine in connection with thyroproteid.

At the French Congress of Neurologists, held at Bordeaux August 1st to 7th, Brissaud[2] opened a discussion on the thyroid gland and Graves' disease. He stated that clinical experience teaches us that certain subjects, who have long been afflicted with simple goitre, may present almost the complete clinical picture of exophthalmic goitre. Impairment of the thyroid secretion by a recent or old standing glandular lesion may, therefore, determine Graves' disease; but it is evident that the toxin in circulation becomes fixed, or localises its effects in the region of the medulla oblongata and pons varolii. This is equivalent to the proposition that the cause of the disease is thyroid intoxication, but the cause of the symptoms is a medullo-pontal localisation of the thyroid poison.

Lesions of the thyroid gland, remarked Brissaud, are unquestionably constant in exophthalmic goitre, but are not specific; they vary widely in different cases, according to circumstances. The hypertrophy of the gland is variable, and, what is more, is not proportional to the severity of the symptoms. It is the result of two widely different lesions: (1) Cystic formations; (2) a sort of hypertrophic cirrhosis, consisting in multiplication of the follicles, or thyroid granules, in the midst of a more or less dense tissue of interstitial sclerosis. These vesicular formations, which are disseminated in the intralobular connective tissue of exophthalmic goitres, are capable of secreting a thyroid juice, the total quantity of which may be considerable, without their epithelium being that of the adult state. This would, indeed, explain hyperthyroidation; but it is absolutely illogical to conclude that Graves' disease is of the thyroid nature, merely from the fact that the thyroid gland presents other lesions than those of the vascular apparatus. This point he had investigated by examining carefully the structure of twenty-five thyroid glands taken without selection from adult subjects who never had manifested the least symptom of Graves' disease. He arrived at the conclusion that the thyroid gland is never healthy in an adult who has succumbed to a chronic disease.

Renaut[3] (Lyons) has found that the thyroid gland in every case of Graves' disease that he has examined, whether hypertrophied or not, is invariably diseased in the same manner. He constantly met with granules and choked epithelial tubules connecting them, lined by a thin connective tissue of new formation, forming a layer of endothelioid corpuscles. A strong impregnation with nitrate of silver set them off with all their branches. These do not exist on the surface of the granules from a healthy thyroid of a dog, sheep, or child. Moreover, the interacinous tissue is not composed of areolar tissue, for when the ordinary methods of investigation are employed the latter no longer contain the normal lymphatic spaces, but are more or less distinctly injected with a colloid substance identical with that of the granules. In a healthy dog when the thyroid gland is injected with osmio-picro-argentic mixture, so as to fill up the lymphatic spaces this penetrates all the lymphatic channels. Each granule is surrounded by enormous lymphatic vessels. In the thyroid gland of exophthalmic goitre he always found that the interstitial, impregnating, and fixative injection developed to the utmost in the interlobular spaces; but in the interior of the lobules not a single lymphatic penetrates. The characteristic lesion then appears to be that the whole of the interlobular lymphatics are destroyed, the thyroid granules at the circumference alone discharging their colloid substance into the interlobular spaces, the only exit still open. At the centre, the veins form the only remaining avenue of escape, and this is largely utilised, as evidenced from the fact that the venous capillaries have frequently attained an enormous size, the veins sometimes have been so distended as to have ruptured. The thyroid secretion, therefore, is discharged through the lymphatics at the margin of the lobules, but at the centre exclusively and directly through the veins.

Renaut has further noted that in staining sections with eosin, the colloid substance lies in the marginal granules, and in the interlobular lymphatics, while in the central granules the stain is less marked, and in the granules of new formation, in the centre of the lobule it is usually *nil*. Now, in a human fœtus of about the third month the lobules of the thyroid have already developed a certain number of granules. The contents of the latter consist solely of a glistening substance, which eosin does not stain pink, though exceptionally a granule is found which stains like those of the adult gland. The number of these staining granules rapidly increases with the age of the fœtus. The thyroid epithelium, therefore, when young, secretes a peculiar substance, not stained by eosin, which he describes as thyromucoin; when the epithelium has reached the adult stage, and acquired its normal lymphatic-vascular arrangement, it secretes another substance, which Renaut terms thyrocolloin, the histo-chemical composition of which is that of thyroid colloid substance such as we know it.

Thyromucoin is the direct product of the secretory activity of the thyroid epithelium. It is seen to form in the shape of refractile bullæ. Thyrocolloin is the result of secondary reactions in the small glandular cavity formed by each thyroid granule at the stage of maturity of the thyroid secretion, produced in the natural course of events in all granules normally provided with lymphatic excretory ducts. But, as has been stated, thyrocolloin is not found except in the marginal granules in exophthalmic goitre. Where there are granules of new formation, the product of secretion consists solely of thyromucoin.

Renaut explains the occurrence of Graves' disease on the assumption that the central nervous system presides over the secretion of the gland, and for this regulation there must be centre, and we are justified in affirming that it is situated in the medullo-pontal region. A *primum movens* which is as yet undetermined, but perhaps variable—it may be the result of microbial action, auto-intoxication, extension of pre-existing neuraxial lesions, or shock—produces hyperactivity of the thyroid gland through intervention of the nervous system. Hyperthyroidation commences slowly and sluggishly at first. There is then too much thyroproteid to destroy in the lymphatic canals, and premonitory functional disturbances set in, such as emotivity, anxiety as to surroundings, slight tremor, and glistening of the eyes, which have been observed in experimental hyperthyroidation, and which are rarely absent at the commencement of Graves' disease if carefully observed. This is the inaugural stage of the disease, and also of the fever at the functional period. There is as yet no lesion, but the lesion is being prepared by the reaction of the tissues to the abnormal development of functional activity.

He is convinced that the lesion itself is determined by the hyperthyroidation, which sets up in the gland a sub-inflammatory condition, then thyroiditis, with return of the fœtal state of the secretory elements, and ultimately destruction of the intralobular lymphatics, due to peri-acinous sclerosis. The moment the normal paths of excretion of the thyrocolloin are definitely blocked at the centre of each lobule the period of thyroid intoxication necessarily begins. The product of an abnormal fœtal secretion, the special toxicity of which needs to be investigated, enters upon the scene. Renaut presumes true thyromucoin acts first on the nervous system. He holds it responsible not only for the fever, but for the whole syndrome of Graves' disease.

Whatever the original cause, he holds that there is a period of intoxication of the nervous system and entire organism; after this follows a period of toleration or death.

Gley[4] reminded the meeting that he had proved years ago that the blood of thyroidectomised animals was toxic; the thyroid gland, therefore, must possess antitoxic properties. But Graves' disease cannot be explained on the assumption of exaggeration of the thyroid secretion, for there are cases in which injections of thyroid juice do not appear to have added to the severity of the disease, and in some cases of partial extirpation the disease has been in no way altered. Just as plausible reasons might be adduced to explain Graves' disease by insufficient thyroid secretion. All the accessory symptoms, tremor, contractures and convulsions, paralyses, respiratory, digestive, ocular, and other symptoms are observed in thyroidectomised dogs. The exophthalmos and tachycardia might be accounted for by the goitre determining compression of the cervical sympathetic and pneumogastric nerves. A third hypothesis possible is, that the symptoms of Graves' disease are due to the formation of abnormal toxic products in the changed thyroid gland.

The experimental evidence of the effect of artificial hyperthyroidation on the structure of the gland, as observed by Enriquez and Ballet,[5] becomes a matter of the greatest interest in connection with the pathological observations of Renaut, Brissaud, and Edmunds.[6] Experimentally they have endeavoured to induce hyperthyroidation by three different procedures: grafting, ingestion, and injection of thyroid extracts, with the following results: (*a*) Age was an important factor in every case, very young dogs succumbing to doses which older ones bore well. (*b*) Hyperthyroidation by ingestion of sheep's thyroids was practised in six dogs. In no case, although one of these animals absorbed 800 lobes in forty days, did death ensue. This is a fact of importance, seeing that it does not accord with the serious symptoms observed in man, in some fatal cases, after the ingestion of thyroid gland of sheep.

The symptoms observed after ingestion of the thyroid gland closely resembled those constituting the clinical symptom of Graves' disease. In not a single instance, however, did they meet with any modification in size of the thyroid gland.

Hyperthyroidation by means of subcutaneous injections of thyroid extract was practised on twelve dogs. By this method hyperthyroidation was found to be much more constant, effective, and rapid, and fatal results occurred in five cases. The dose of the extract appeared to have less influence on the gravity of the symptoms than the age of the animal, young animals being profoundly affected by relatively small doses.

The injections, which were invariably practised at a distance from the thyroid gland, were followed by important changes in the thyroid gland, viz., enlargement of the gland and histological changes, similar to those described by Renaut—obliteration of the intralobular lymph paths, associated with exaggerated development of the perilobular lymph paths. There were also (according to Renaut, who examined his sections) a complete substitution of granular tissue for that of the normal gland substance in all the lobules. They add that one point of great interest lies in the intense inflammatory reaction determined in the thyroid gland by injection at a distance of thyroid extract. This fact may be taken to indicate that the functional antitoxic action of the thyroid gland, as at present understood, takes place within the gland itself and not in the circulation. Moreover, the sclerotic transformation of the gland, with complete destruction of the alveoli and epithelial cells, may also explain certain clinical cases, in which symptoms of myxœdema have followed Graves' disease. In the opinion of these experimenters, the primary phenomenon in Graves' disease consists in exaggeration of the functional activity of the thyroid gland, which secretion stimulates the nuclei in the medulla, or accessorily in the spinal cord, which preside over the symptomatology of Graves' disease; and secondly, the lesions in the gland are determined by hyperthyroidation. Finally, these observers tried the effect of injecting blood-serum from thyroidectomised dogs into certain patients suffering from Graves' disease, and the results in nine cases were most gratifying.

We may refer to Hurthle's[7] observations, who states that the colloid substance in the follicles is produced by the protoplasm of the epithelial cells. The secretion of the gland consists in the formation of colloid matter, and this can be increased by removal of the greater part of the gland, and also by icterus produced by ligature of the bile ducts. Matton[8] refers to a case which presented simultaneously symptoms of Graves' disease and hypertrophic cirrhosis of the liver with jaundice.

Other two records bearing on the question of inflammatory changes in the thyroid are of special interest in connection with Ballet and Enriquez' experiments. Engle-Renners[9] have observed enlargement of the thyroid in 130 cases of early syphilis. In no case were symptoms of Graves' disease observed, though in one myxœdema occurred and was cured by the use of mercury. Reinhold[10] reports a case in which acute stumitis occurred in influenza. The stumitis subsided, but three months later the patient was suffering from Graves' disease. The writer could only find one other similar case recorded.

Béclère[11] reports a case of a woman, age 31, whose case furnished an instance of the cure of myxœdema by ingestion of thyroid gland. In consequence of a misunderstanding she took at the outset as much as 92 grammes in eleven days, with resulting thyroid intoxication. But in addition to tachycardia, rise of temperature, insomnia, agitation-polyuria, glycosuria, albumenuria, and incomplete paraplegia, there was hastened respiration, transient tremor of the arms, exophthalmia, and brilliancy of the eye. Another peculiarity was that, although the patient was not in the least neuropathic before treatment, she developed transient aphasia with monoplegia and anæsthesia of the right arm, manifestly of hysterical nature.

**The Thymus Gland and Graves' Disease.**—Dr. Owen,[12] in December, 1893, recorded a marked case of exophthalmic goitre of twenty years' duration, for which thyroid feeding had been prescribed, resulting in the disappearance of all the symptoms of Graves' disease, but on re-investigation he found that the patient had been supplied with thymus gland and not thyroid at all. In January, 1894, the thymus was discontinued for a time, though against the wish of the patient, who attributed his relief to its administration. In course of time the Graves' disease symptoms began to return, viz., palpitation and increased size of the thyroid gland; and after March 20th the thymus gland was resumed with great benefit, and on July 25th, 1894, when the patient was last seen, the improvement in his condition was very remarkable, and he had gained in weight and strength, and he had been free from palpitation for six months. The pulse, which was formerly constantly over 120, was then 72, the eye symptoms had disappeared, the thyroid swelling was no longer present, melancholia was replaced by a feeling of well-being. He had been taking one lobe of neckberg—the cervical portion of the thymus—three or four times a week. The patient stated that he had several times discontinued the gland for a time, but finding himself getting worse had resumed it, always with immediate benefit, and during the latter two months had taken one lobe a week. Mikulicz[13] suggests that two different substances may be contained in the thyroid—one which prevents myxœdema, and which can only be supplied by the thyroid gland; the other, which is useful in cases of goitre, and which may also be obtained from the thymus gland. Mikulicz himself has employed thymus feeding in eleven cases, ten of goitre and one of Graves' disease, and, as far as he can see, the result is similar to that obtained by thyroid feeding. He always gave 10 to 25 grammes of raw sheep's thymus, in gradually-increasing doses, and about three times weekly. Out of ten cases of goitre one was cured, six decidedly and two somewhat improved, one only being unaffected. In the case of Graves' disease, the subjective symptoms, exophthalmos and tachycardia, were all diminished, but the goitre and tremor remained practically the same.

Other cases are recorded by Cunningham[14], in which thymus feeding was employed in Graves' disease. The first case was that of a young lady, age 20, who had suffered from Graves' disease for some time. When first seen the pulse rate was 124 a minute, and exophthalmos, insomnia, mental depression, excessive sweating on the right side at night, tremor, &c., were present. At first thyroid tablets were given, but these only made her worse. Fresh lamb's thymus was substituted, and taken broiled. In ten days there was some improvement, and six months later the patient reported perfect health; pulse 72, and the goitre and exophthalmos had disappeared. In two other cases there was marked improvement; in one, after taking freshly-cooked lamb's thymus, and in the other less rapid improvement after taking twelve to fifteen five-grain tabloids of thymus daily for a week. As bearing on the function of the thymus, the writer states that he has fed thyroidectomised dogs on thymus gland without apparently hastening or delaying the appearance of the cachexia otherwise than if the animals had been fed on so much ordinary cooked meat, although Breisacher has pointed out that when thyroidectomised dogs are fed on meat that has been thoroughly extracted by boiling, the cachexia is delayed, and that in dogs fed on the bouillon from the meat the cachexia develops with unusual rapidity.

Furthermore, there are the interesting observations of Ludwig Hektoen[15] on a case of hyperplastic persistent thymus in exophthalmic goitre in a female aged 20, a case of which was first seen November 29th, 1892, with well marked prominence of the eyes, drooping of left lid, uniformly enlarged thyroid, tumultuous heart, and with the tissue over the anterior aspect of the lower third of each leg swollen and elastic like myxœdema. She also had frequent spells of constant vomiting. The symptoms became more pronounced and emaciation progressive till she died on January 20th, 1893. A report of the post-mortem examination is given in detail. In the thyroid gland the stroma was increased in greater proportion than the glandular part, the stroma being richly cellular, but in places less vascular than normal; certainly the vessels were not large or numerous. The markedly proliferated cells of the tubules were more ovoidal than cuboidal, never cylindrical, and in only a few follicles was there any colloidal material. The thymus sections presented the same appearance as that of an active thymus in a very young person. The author cites other instances of persistent thymus in exophthalmic goitre recorded by Mosler,[16] Lasvenes,[17] Johnstone,[18] Spencer,[19] White[20] (two cases), Möbius,[21] Reymond,[22] Koppen,[23] Clarke,[24] Hilton Fagge,[25] Marie[26] (six cases), though on account of the indefinite descriptions the author admits that some may be instances of persistence of the retrosternal fat body that has been shown to preserve the form of the atrophied thymus. Another instance of persistent thymus is given by Murray.[27]

[1] The Hospital, Aug. 24, 1895, p. 359. [2] Med. Week, p. 388. [3] Ibid. [4] Ibid. [5] Ibid. [6] Hospital, Aug. 24, 1895, p. 360. [7] Pfluger's Archiv. fur die gesammte Phys. Bd. 56, Journ. Laryng. Nov. 1895. [8] Med. Week, 1895, p. 394. [9] Jahr. des Hamburg, Staabkrank, Jahr. 1892-93, Journ. of Laryn. xii., 1894. [10] Munich. Med. Woch., 1893, No 23., Journ. of Lar. xii., 1894. [11] Med. Soc. des Hop., Oct. 12, 1894. [12] Brit. Med. Journ., Feb. 16, 1895. [13] Berlin Klin, Woch., April 22, 1895. [14] New York Med. Rec., June 15, 1895. [15] Med. Mag., Sep. 1895, p. 584. [16] Krankengeschicht, Griefswald, 1889. [17] Thèse de Paris, 1891. [18] Journ. of Mental. Science, 1884, p. 521. [19] Trans. Lond. Path. Soc., 1891. [20] Brit. Med. Journ., 1880 and 1886. [21] Cited. Schmidts. Jahr. B. 193, p. 25. [22] Bull. de Soc. Anat. de Paris, sec. 5, vol. vii., No. 18. [23] Neurol Central, 1892, No. 19. [24] Bristol Med. Journ., 1887, No. 15. [25] Medicine p. 1,012. [26] Gaz. des. Hôp., Feb. 21, 1893. [27] Med. Week, No. 33, 1895, p. 393.

